# Landscape analysis and overview of the literature on oxidative stress and pulmonary diseases

**DOI:** 10.3389/fphar.2023.1190817

**Published:** 2023-05-26

**Authors:** Xin Liu, Xiaofan Wang, Jing Chang, Hongmin Zhang, Pengxiu Cao

**Affiliations:** Ministry of Education Key Laboratory of Molecular and Cellular Biology, Hebei Key Laboratory of Animal Physiology, Biochemistry, and Molecular Biology, College of Life Sciences, Hebei Normal University, Shijiazhuang, Hebei, China

**Keywords:** oxidative stress, inflammation, mitochondria, antioxidants, pulmonary disease

## Abstract

Oxidative stress is caused by an imbalance in oxidant/antioxidant processes and is a critical process in pulmonary diseases. As no truly effective therapies exist for lung cancer, lung fibrosis and chronic obstructive pulmonary disease (COPD), at present, it is important to comprehensively study the relationship between oxidative stress and pulmonary diseases to identify truly effective therapeutics. Since there is no quantitative and qualitative bibliometric analysis of the literature in this area, this review provides an in-depth analysis of publications related to oxidative stress and pulmonary diseases over four periods, including from 1953 to 2007, 2008 to 2012, 2013 to 2017, and 2018 to 2022. Interest in many pulmonary diseases has increased, and the mechanisms and therapeutic drugs for pulmonary diseases have been well analyzed. Lung injury, lung cancer, asthma, COPD and pneumonia are the 5 most studied pulmonary diseases related to oxidative stress. Inflammation, apoptosis, nuclear factor erythroid 2 like 2 (NRF2), mitochondria, and nuclear factor-κB (NF-κB) are rapidly becoming the most commonly used top keywords. The top thirty medicines most studied for treating different pulmonary diseases were summarized. Antioxidants, especially those targeting reactive oxygen species (ROS) in specific organelles and certain diseases, may be a substantial and necessary choice in combined therapies rather than acting as a single “magic bullet” for the effective treatment of refractory pulmonary diseases.

## Introduction

Oxidative stress is an upstream event leading to multiple diseases, including cancer, cardiovascular diseases, neuron degenerative diseases and pulmonary diseases (Klaunig, 2018; van der Vliet et al., 2018; Singh et al., 2019; Steven et al., 2019). In organisms, the production of free radicals, including reactive oxygen species (ROS) and reactive nitrogen species (RNS), is mediated by extrinsic factors (drugs, radiation, inorganic particles, and tobacco smoking (Tostes et al., 2008; Helmig et al., 2018; Owusu et al., 2020; Uchiyama et al., 2020)) or endogenous sources (mitochondria, oxidative enzymes, and peroxisomes) (Dan Dunn et al., 2015; Forrester et al., 2018). ROS include superoxide radical anions (O_2_^−^), hydroxyl radicals (OH), hydrogen peroxide (H_2_O_2_) and hypohalite radicals (HOO.). NO is the main RNS synthesized by numerous cells and is central to the formation of other RNS (Martinez and Andriantsitohaina, 2009). The antioxidant system, including antioxidative enzymes (such as SOD, catalase, glutathione peroxidase and glutathione S-transferase) (Margis et al., 2008; Weydert and Cullen, 2009; Chatterjee and Gupta, 2018) and nonenzymatic antioxidants (such as N-acetylcysteine, glutathione, and vitamin E) (Schmitt et al., 2015; Lee and Han, 2018), helps to remove excessive free radicals by acting on them directly or indirectly by consuming substances that produce free radicals. Excess free radicals can cause oxidative stress if their levels exceed the antioxidation limits both *in vivo* and *in vitro* (Pizzino et al., 2017).

Pulmonary diseases are one of the main factors contributing to the morbidity and mortality of older individuals, who experience these diseases at a higher prevalence. Due to smoking, second-hand smoke or air pollution, the occurrence of pulmonary diseases in young persons is increasing annually. Unfortunately, no truly effective interventions are currently available for some lung diseases, such as lung cancer, chronic obstructive pulmonary disease (COPD), and lung fibrosis. Therefore, it is important to elucidate their mechanisms to identify better therapeutic strategies to cure patients with these diseases.

Most pathogenicity factors, including viral and bacterial infections, air pollution, occupational exposure, smoking, and aging, contribute to pulmonary diseases through oxidative stress (Cheresh et al., 2013). For instance, exposure to excess fine particulate matter, respirable fibers, inhalable quartz and metal powders, ozone, and vehicle exhaust from the environment causes oxidative stress, which initiates pulmonary inflammation, carcinogenesis, and fibrosis (Valavanidis et al., 2013; Han et al., 2023). Oxidative stress induced by cigarette smoke leads to mitochondrial dysfunction of human bronchial epithelial cells, increased risk of emphysema through upregulation of MMP-9 activity, and EGFR activation, which results in the decrease of lung functions and hypersecretory diseases of the airways (Malinska et al., 2018; Muratani et al., 2023; Staitieh et al., 2023). This study aims to provide the first in-depth quantitative and qualitative bibliometric analysis of publications related to oxidative stress and pulmonary disease over time from 1953 to 2022 and to identify the major contributors, emerging mainstream research themes, and therapeutic drugs. The authors, institutions, countries, and journals with the highest output for each period were analyzed; the keywords with occurrences in the titles and abstracts of most publications were identified and provide important information on the major mechanisms underlying the function of oxidative stress in pulmonary diseases. Furthermore, the developing trend of publications in major research categories and annual publications on major pulmonary diseases were analyzed. Moreover, potential therapeutic drugs are summarized. Hopefully, by presenting a comprehensive view of recent research accomplishments on the relationship between oxidative stress and pulmonary diseases, this paper provides novel insights into the development of truly effective therapies for refractory pulmonary diseases.

## Materials and methods

An advanced search in the Web of Science (WOS) electronic databases was performed by “Topic search” to obtain the whole body of publications concerning both oxidative stress and pulmonary diseases (33 kinds in total) from 1953 to 2022 or four separate time periods, including from 1953 to 2007, 2008 to 2012, 2013 to 2017 and 2018 to 2022. The retrieval pattern was as follows: TS = (“Oxidative Stress*”) AND TS=(“corpulmonale” OR “lung cancer” OR “lung carcinoma” OR “pulmonary carcinoma” OR “carcinoma of lung*” OR “carcinoma of the lung*” OR “pulmonary sarcoidosis” OR “lung sarcoidosis” OR “pulmonary artery hypertension” OR “pulmonary arterial hypertension” OR “pulmonary hypertension” OR “lung artery hypertension” OR “lung arterial hypertension” OR “lung hypertension” OR “pulmonary embolism” OR “lung embolism” OR “pulmonary thromboembolism” OR “lung thromboembolism” OR “COPD” OR “chronic obstructive pulmonary disease*” OR “lung infection*” OR “pulmonary infection*” OR “infectious lung disease*” OR “infectious pulmonary disease*” OR “pulmonary mycosis” OR “lung mycosis” OR “pulmonary candidiasis” OR “lung candidiasis” OR “pulmonary aspergillosis” OR “lung aspergillosis” OR “pulmonary cryptococcosis” OR “lung cryptococcosis” OR “pulmonary tuberculosis” OR “lung tuberculosis” OR “pneumonia” OR “pulmonary abscess” OR “lung abscess” OR “bronchitis” OR “acute tracheobronchitis*” OR “alveolitis” OR “asthma” OR “bronchiectasis” OR “emphysema” OR “pulmonary bullae” OR “lung bullae” OR “pneumothorax” OR “pleural effusion” OR “hypoxemia” OR “pulmonary lymphangioleiomyomatosis” OR “lung lymphangioleiomyomatosis” OR “pulmonary edema” OR “lung edema” OR “alveolar proteinosis” OR “lung injury*” OR “pulmonary injury*” OR “dyspnea” OR “ventilation dysfunction” OR “acute respiratory distress syndrome*” OR “respiratory failure” OR “sleep apnea” OR “pulmonary fibrosis” OR “pulmonary fibrogene*” OR “lung fibrosis” OR “lung fibrogene*” OR “Cystic fibrosis” OR “idiopathic pulmonary fibrosis” OR “pneumoconiosis” OR “BOS” OR “bronchiolitis obliterans syndrome*” OR “lung disease*” OR “pulmonary disease*”). After the retrieval results were shown, by clicking on “Create Citation Report” and then “Analyze”, the top productive authors, institutions, countries/regions, and journals were displayed.

The keywords over four periods from 1953 to 2007, 2008 to 2012, 2013 to 2017 and 2018 to 2022 were obtained by importing all the publications on oxidative stress and pulmonary diseases during the corresponding period from WOS electronic databases into VOSviewer1.6.18, and the most frequent 25 keywords during each period were identified.

Bubble diagrams were generated by importing the publications on oxidative stress and pulmonary diseases during different time periods into VOSviewer1.6.18 for all keyword analyses, and those appearing in at least 2% of publications were included in the bubble diagrams.

The numbers of publications related to iron, mitochondria, or inflammation in four time periods were obtained by adding the retrieval conditions of TS = (“inflammat*”), TS = (“iron” or “Ferroptosis”), or TS = (“mitochondria” or “mitochondrial” or “mitochondrion”), respectively, to the previous retrieval patterns in the advanced search through the WOS electronic databases.

The publication number on each kind of pulmonary disease related to oxidative stress was obtained through the advanced search in the WOS electronic databases by using the retrieval pattern of TS = (“Oxidative Stress*”) and TS = (“the corresponding disease”).

After importing all the publications on oxidative stress and pulmonary diseases into VOSviewer1.6.18, the drugs that appeared as keywords in more than 20 publications in VOSviewer1.6.18 were selected and ordered by their publication numbers. The numbers of publications on the top thirty drugs on eight common pulmonary diseases were analyzed in VOSviewer1.6.18 by the retrievals of TS = (“Oxidative Stress*") and TS = (“the corresponding disease”) through the advanced search in the WOS electronic databases.

## Results

### Publications on oxidative stress and pulmonary diseases from each time period were analyzed

First, we searched all publications concerning both oxidative stress and lung diseases from 1953 to 2022 to analyze the roles of oxidative stress in pulmonary diseases. As a result, a total of 31,373 publications from 1953 to 2022 were obtained. Among them, the top twenty authors, institutions, countries/regions, and journals with the highest productivity were retrieved, and this information is shown in Table 1. In the retrieval record, the first names and middle names of authors were initialized by WOS; therefore, some of the author’s name abbreviations may represent multiple author identities. It was found that the most prolific author name “Wang Y” by the original search data corresponds to multiple author identities, such as “Wang Yan”, “Wang Ye” and “Wang Yong”. By excluding the names corresponding to multiple identities, we found that the most prolific author was BARNES PJ in the National Heart, Lung, and Blood Institute, University of London, United Kingdom [*n* = 179/0.57%, citation per publication (CPP) = 130.56]. Table 1 shows that the most productive institution, countries/region, and journal are the University of California System (*n* = 716/2.28%, CPP = 59.48), the United States (*n* = 8,800/28.04%, CPP = 56.02), and Free Radical Biology and Medicine (*n* = 571/1.82%, CPP = 56.91).

**TABLE 1:** The top 20 authors, institutions, countries/regions, and journals with the highest productivity on publications on oxidative stress and pulmonary diseases from 1953 to 2022. CPP, citation per publication.

Author	Publication number	Proportion (%)	CPP	H-index
a				
Wang Y	434	1.38	24.53	51
Zhang Y	383	1.22	19.71	38
Li Y	313	1.00	24.16	41
Wang J	276	0.88	18.75	36
Zhang J	273	0.87	25.92	37
Li J	270	0.86	23.35	38
Wang X	269	0.86	33.37	39
Liu Y	263	0.84	23.71	42
Li X	249	0.79	22.75	34
Chen Y	228	0.73	36.46	42
Wang L	218	0.70	19.4	34
Zhang X	207	0.66	21.99	35
Zhang L	204	0.65	23.16	35
Wang H	199	0.63	27.17	35
Liu J	188	0.60	18.11	31
Zhang H	181	0.58	25.67	34
Barnes Pj	179	0.57	130.56	83
Li L	163	0.52	20.36	29
Li H	158	0.50	23.23	28
Liu X	154	0.49	16.22	22

Institution	Publication number	Proportion (%)	CPP	H-index
b				
University of California System	716	2.28	59.48	102
Udice French Research Universities	584	1.86	53.67	82
Harvard University	499	1.59	80.85	94
University of Texas System	495	1.58	65.39	84
Egyptian Knowledge Bank Ekb	490	1.56	13.94	39
Pennsylvania Commonwealth System of Higher Education Pcshe	487	1.55	59.19	89
Institut National De La Sante Et De La Recherche Medicale Inserm	485	1.55	43.11	76
Imperial College London	468	1.49	80.15	104
Us Department of Veterans Affairs	378	1.20	60.77	71
Johns Hopkins University	374	1.19	111.93	102
Veterans Health Administration Vha	365	1.16	62.36	71
National Institutes of Health Nih United States	343	1.09	81.7	78
University of Pittsburgh	343	1.09	68.78	84
Chinese Academy of Sciences	329	1.05	35.04	51
Universidade De Sao Paulo	322	1.03	24.18	43
Harvard Medical School	308	0.98	80.87	75
University of Colorado System	292	0.93	58.93	66
Ciber Centro De Investigacion Biomedica En Red	285	0.91	32.54	49
Emory University	274	0.87	37.82	59
University System of Ohio	267	0.85	50.1	59

Country/region	Publication number	Proportion (%)	CPP	H-index
c				
United States	8,800	28.04	56.02	271
China	6,376	20.32	22.37	117
Italy	1,754	5.59	46.16	130
Japan	1,518	4.84	42.08	113
England	1,392	4.44	61.93	149
Germany	1,297	4.13	48.79	109
India	1,254	4.00	25.45	74
South korea	1,128	3.59	27.9	81
Canada	1,096	3.49	47.58	103
Brazil	1,045	3.33	21.42	63
France	1,011	3.22	44.6	93
Turkey	1,005	3.20	20	56
Spain	884	2.82	44.45	92
Taiwan	784	2.50	27.68	69
Australia	771	2.46	36.87	81
United Kingdom	760	2.42	86.91	134
Iran	687	2.19	24.45	60
Netherlands	641	2.04	56.82	94
Poland	560	1.78	28.64	60
Egypt	499	1.59	13.88	39

Journal	Publication number	Proportion (%)	CPP	H-index
d				
Free Radical Biology and Medicine	571	1.82	56.91	85
PloS One	526	1.68	33.58	58
American Journal of Respiratory and Critical Care Medicine	415	1.32	79.9	113
American Journal of Physiology Lung Cellular and Molecular Physiology	411	1.31	51.29	76
European Respiratory Journal	342	1.09	56.25	72
FASEB Journal	341	1.09	15.54	38
International Journal of Molecular Sciences	323	1.03	23.46	46
Oxidative Medicine and Cellular Longevity	314	1.00	26.1	47
American Journal of Respiratory Cell and Molecular Biology	312	0.99	59.66	75
Scientific Reports	303	0.97	22.77	46
The European Respiratory Journal	239	0.76	77.58	71
Antioxidants Basel Switzerland	235	0.75	11.21	27
Antioxidants	234	0.75	11.34	27
Journal of Dairy Science	224	0.71	31.1	44
International Immunopharmacology	222	0.71	22.86	39
Antioxidants Redox Signaling	216	0.69	73.48	69
Respiratory Research	196	0.63	34.78	46
Biomedicine Pharmacotherapy	194	0.62	30.55	32
Frontiers in Pharmacology	181	0.577	14.2	27
Chest	179	0.572	75.73	62

**FIGURE 1:**
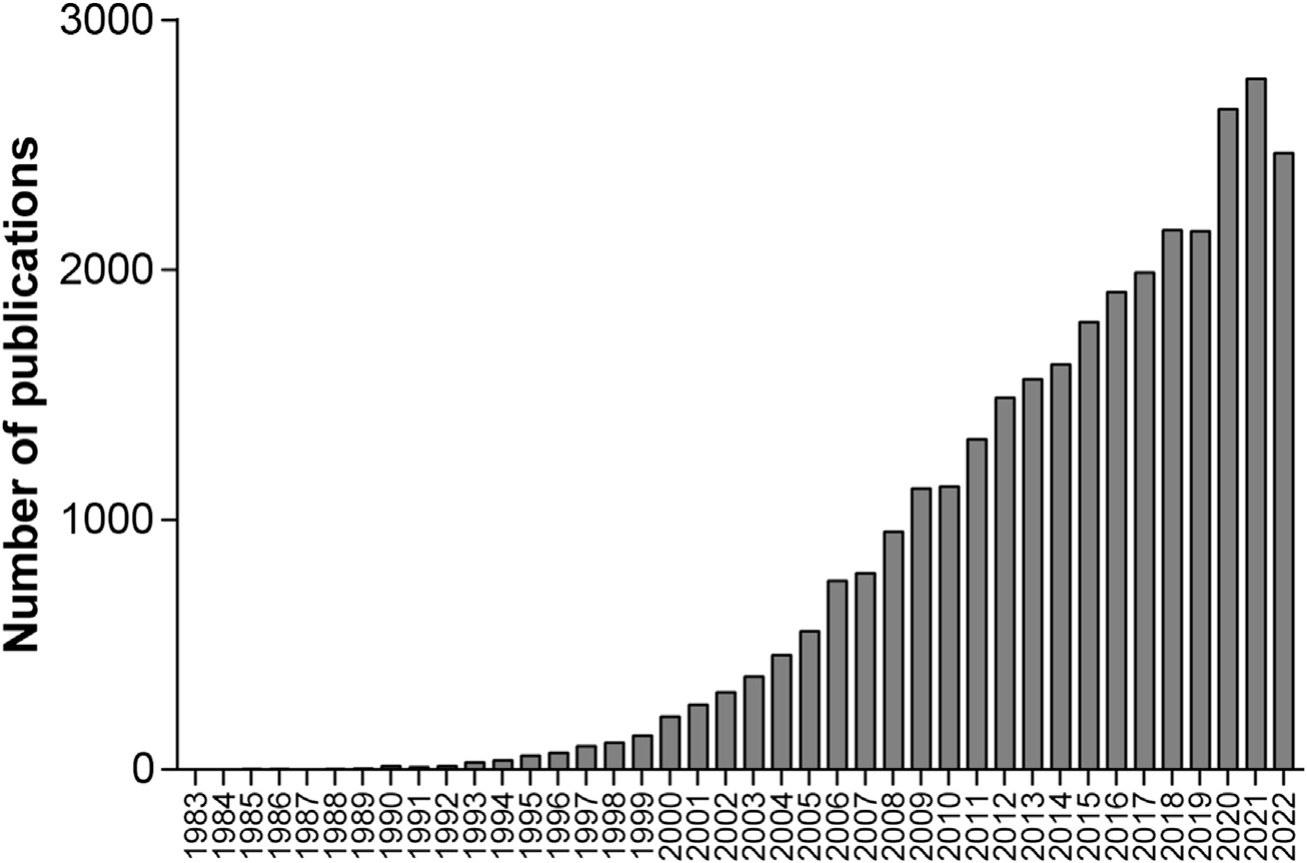
Annual count of publications on oxidative stress and lung diseases from 1983 to 2022.

Among the 31,373 retrieved publications, 29,619 (94.41%) were articles, and 30,738 (97.98%) were written in English. The annual count of these publications shown in Figure 1 indicates that the annual publications in 2009 exceeded 1,000 for the first time. To show the developing trend of the vast amount of research from 1953 to 2022, we separated these 31,373 publications into four groups based on the publication time periods from 1953 to 2007, 2008 to 2012, 2013 to 2017 and 2018 to 2022, which include 4,254, 5,673, 8,471, and 12,975 publications, respectively. Then, we reanalyzed these publications, and the number of papers, English publications, and average citations per publication; the most referenced publication and the top prolific author; and the top five institutions, countries/regions, and journals with the highest productivity are summarized in Table 2.

**TABLE 2:** The information of the publications on oxidative stress and pulmonary diseases during four time periods were shown. CPP, citation per publication.

	1953–2007	2008–2012	2013–2017	2018–2022
a. The count of publications, papers, English publications, average citations per publication, the most referred publication, and the top prolific author of the publications during four time periods were presented.				
Number of publications/number and percentage of papers/number and percentage of English publications	4,254/3,994 (93.9%)/4,061(95.5%)	5,673/5,145(90.7%)/5,500(97.0%)	8,471/7,962(94.0%)/8,326(98.3%)	12975/12518(96.5%)/12851(99.0%)
Average number of citations per publication	80.28	52.94	37.99	14.89
Title, journal, and publication year of the most cited paper	Free radicals in the physiological control of cell function, Physiological Reviews, 2002	Targeting cancer cells by ROS-mediated mechanisms: a radical therapeutic approach? Nature Reviews Drug Discovery, 2009	2014 AHA/ACC/HRS Guideline for the Management of Patients with Atrial Fibrillation, JOURNAL OF The American College of Cardiology, 2014	Oxidative stress, aging, and diseases, Clinical Interventions in Aging, 2018
Most prolific author (number of publications/percentage of publications/CPP/H-index/With exclusion of more prolific author names with multiple identities	Barnes Pj (72/0.017%/176.63/59/No)	Rahman I (46/0.811%/106.52/35/No)	Wang Y (106/1.251%/37.08/38/No)	Wang Y (278/2.143%/14.65/34/No)

b. Top five institutions and countries/regions with the highest productivity of the publications during four time periods were presented.				
Top five most productive institutions (number of publications/percentage of publications/citations per publication)	University of California System (147/3.46%/91.22/65), Imperial College London (127/2.99%/133/70), Pennsylvania Commonwealth System of Higher Education Pcshe (93/2.19%/110.51/55), Udice French Research Universities (93/2.19%/85.84/39), University of Texas System (86/2.02%/68.13/44)	Udice French Research Universities (142/2.50%/79.2/53), University of California System (139/2.45%/87.33/56), University of Texas System (130/2.29%/109.58/50), Harvard University (121/2.13%/72.31/50), Institut National De La Sante Et De La Recherche Medicale Inserm (108/1.90%/60.53/48)	University of California System (194/2.29%/57.3/58), Udice French Research Universities (163/1.92%/44.2/46), Harvard University (146/1.72%/88.55/55), Institut National De La Sante Et De La Recherche Medicale Inserm (145/1.71%/42.16/43), University of Texas System (139/1.64%/52.73/45)	Egyptian Knowledge Bank Ekb (324/2.50%/8.48/24), University of California System (236/1.82%/25.17/37), Chinese Academy of Sciences (213/1.64%/17.35/30), Udice French Research Universities (186/1.43%/26.52/33), Institut National De La Sante Et De La Recherche Medicale Inserm (166/1.28%/22.77/32)
Top five most productive countries/regions (number of publications/percentage of publications/citations per publication)	United States (1,599/37.59%/103.85/193), Japan (318/7.48%/68.81/80), England (310/7.29%/106.98/97), United Kingdom (251/5.90%/140.48/96), Italy (242/5.69%/93.62/82)	United States (2001/35.27%/71.36/170), China (439/7.74%/49.61/67), Japan (369/6.50%/52.33/70), Italy (321/5.66%/59.81/76), England (255/4.50%/74.14/80)	United States (2,420/28.57%/51.24/144), China (1700/20.07%/34.63/92), Italy (479/5.66%/45.84/71), Japan (414/4.89%/34.11/60), England (372/4.39%/61.11/76)	China (4,151/31.99%/13.38/74), United States (2,777/21.40%/21.7/99), Italy (711/5.48%/24.15/58), India (667/5.14%/15.63/44), Brazil (500/3.85%/12.96/33)

c. Top five journals with the highest productivity of the publications during four time periods were presented.				
Top five periodicals with the most output (number of publication’s/percentage of publications/CPP/H-index)	American Journal of Respiratory and Critical Care Medicine (159/3.74%/123.4), Free Radical Biology and Medicine (140/3.29%/83.77), Free Radical Biology Medicine (139/3.27%/84.37), American Journal of Physiology Lung Cellular and Molecular Physiology (125/2.94%/73.74), European Respiratory Journal (96/2.26%/112.88)	Free Radical Biology Medicine (174/3.07%/69.3/55), Free Radical Biology and Medicine (173/3.05%/69.68/55), American Journal of Respiratory and Critical Care Medicine (107/1.89%/75.52/57), American Journal of Physiology Lung Cellular and Molecular Physiology (90/1.59%/58.49/43), European Respiratory Journal (90/1.59%/61.02/41)	Plos One (377/4.45%/35.06/52), Scientific Reports (119/1.41%/38.21/41), American Journal of Physiology Lung Cellular and Molecular Physiology (118/1.39%/42.72/42), Free Radical Biology and Medicine (118/1.39%/54.25/46), Free Radical Biology Medicine (116/1.37%/55.19/46)	International Journal of Molecular Sciences (264/2.04%/17.44/33), Oxidative Medicine and Cellular Longevity (231/1.78%/16.35/31), Antioxidants Basel Switzerland (225/1.73%/11.12/26), Antioxidants (223/1.72%/11.2/26), Scientific Reports (183/1.41%/12.74/24)

### Top keywords with the most occurrences in the publications provided main mechanisms underlying the effects of oxidative stress on pulmonary diseases

To identify the important molecular events regarding oxidative stress in pulmonary diseases, we searched for the keywords by importing the publications into VOSviewer1.6.18. The most frequent 25 keywords in the publications over five periods from 1953 to 2022, 1953 to 2007, 2008 to 2012, 2013 to 2017 and 2018 to 2022 are indicated in Table 3. Most of the 25 keywords can be sorted into 3 categories. The first category includes oxidative stress or molecules that are closely related, such as oxidative damage, ROS, lipid peroxidation, SOD, MDA, glutathione, glutathione peroxidase, nitric oxide, and catalase. The second category comprises lung diseases or factors that are closely connected, including COPD, lung injury, asthma, lung cancer, obstructive sleep apnea, cystic fibrosis, hypertension, cigarette smoke, particle, lipopolysaccharide (LPS), biomarker, bronchoalveolar lavage fluid, and age. The third category includes molecules, organelles, or cell functions that play a role in both oxidative stress and pulmonary diseases, including NRF2 (nuclear factor, erythroid 2 like 2), nuclear factor-κB (NF-κB), TNF-alpha, cytokines, DNA damage, macrophages, heme oxygenase, endothelial dysfunction, inflammation, apoptosis, cell death, and hypoxia. Among the top 25 keywords of all four time periods, ROS, COPD, asthma, lung injury, cancer, biomarker, smoke, inflammation, apoptosis, caspase, catalase, glutathione, MDA, SOD, and TNF-alpha appeared during at least three periods. In a comparison of the top 25 keywords during different time periods, NRF2, NF-κB, and LPS first arose from 2013 to 2017 and have been reported after, indicating that research on these three terms involves a comparatively novel mechanism, new interests or reagents used by scientists conducting research on oxidative stress and pulmonary diseases starting in 2013.

**TABLE 3:** The 25 most frequent keywords with publication number and the proportion of the publications on oxidative stress and pulmonary diseases in the corresponding time period.

Time period	Keywords (publication number/percentage in all publications of the time period)
1953–2007	injury (612/14.4%), model (565/13.3%), asthma (449/10.6%), COPD (398/9.4%), lipid peroxidation (351/8.3%), apoptosis (343/8.1%), ROS (308/7.2%), macrophage (272/6.4%), glutathione (269/6.3%), cytokine (266/6.3%), cancer (246/5.8%), oxidative damage (232/5.5%), DNA (211/5.0%), catalase (210/4.9%), SOD (206/4.8%), particle (203/4.8%), MDA (189/4.4%), glutathione peroxidase (183/4.3%), airway inflammation (179/4.2%), heme oxygenase (174/4.1%), biomarker (174/4.1%), cigarette smoke (167/3.9%), imbalance (167/3.9%), acute lung injury (166/3.9%), cystic fibrosis (165/3.9%)
2008–2012	age (437/7.7%), hypertension (389/6.9%), biomarker (323/5.7%), SOD (267/4.7%), MDA (255/4.5%), cell death (254/4.5%), smoking (241/4.2%), catalase (233/4.1%), cardiovascular disease (224/3.9%), morbidity (224/3.9%), glutathione peroxidase (219/3.9%), obstructive sleep apnea (212/3.7%), polymorphism (203/3.6%), acute lung injury (202/3.6%), cancer cell (194/3.4%), intermittent hypoxia (191/3.4%), phosphorylation (191/3.4%), caspase (180/3.2%), bronchoalveolar lavage fluid (172/3.0%), cytotoxicity (166/2.9%), endothelial dysfunction (153/2.7%), isoprostane (153/2.7%), TNF-alpha (132/2.3%), obesity (129/2.3%), tumor (129/2.3%)
2013–2017	apoptosis (1,113/13.1%), ROS (953/11.3%), COPD (753/8.9%), cancer (720/8.5%), asthma (662/7.8%), biomarker (655/7.7%), lung injury (652/7.7%), SOD (652/7.7%), age (611/7.2%), MDA (509/6.0%), lung cancer (495/5.8%), TNF-alpha (491/5.8%), mortality (486/5.7%), glutathione (481/5.7%), acute lung injury (417/4.9%), NRF2 (417/4.9%), caspase (405/4.8%), cigarette smoke (404/4.8%), serum (370/4.4%), nitric oxide (357/4.2%), NF-κB (352/4.2%), catalase (344/4.1%), bronchoalveolar lavage fluid (319/3.8%), cardiovascular disease (309, 3.6%) LPS (248, 2.9%)
2018–2022	apoptosis (1980/15.3%), cancer (1912/14.7%), ROS (1717/13.2%), lung injury (1708/13.2%), infection (1,143/8.8%), pathogenesis (1,131/8.7%), drug (1,068/8.2%), COPD (1,044/8.0%), biomarker (1,038/8.0%), metabolism (1,033/8.0%), TNF-alpha (1,007/7.8%), SOD (999/7.7%), MDA (901/6.9%), mortality (901/6.9%), NRF2 (853/6.6%), MDA (805/6.2%), hypoxia (759/5.8%), LPS (724/5.6%), NF-κB (724/5.6%), cancer cell (699/5.4%), inflammatory cytokine (673/5.2%), glutathione (667/5.1%), macrophage (659/5.1%), caspase (651/5.0%), death (644/5.0%)

### The developing trend of publications in major research categories was demonstrated

**FIGURE 2:**
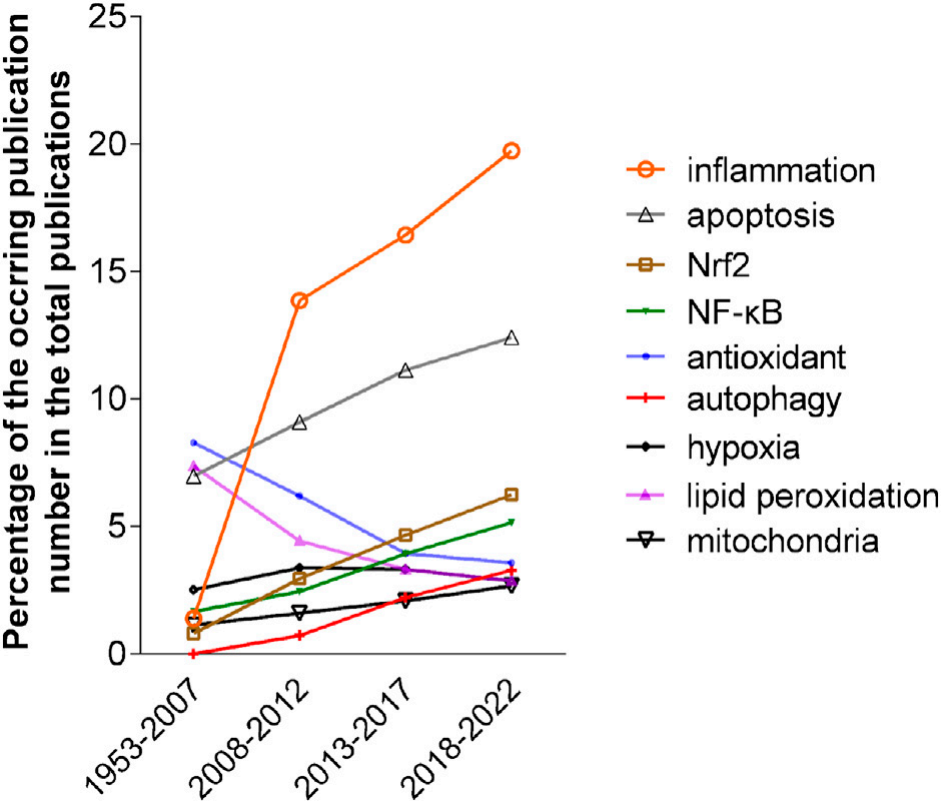
The occurrence trend of eight keywords in four periods, 1953–2007, 2008–2012, 2013–2017 and 2018–2022. The occurrences were calculated as the percentage of the number of publications of each keyword in the total number of publications in the corresponding periods.

VOSviewer identified 258 keywords that appeared in more than 2% (*n* = 260) of 12,975 publications between 2018 and 2022. Among them, except for those of oxidative stress and a few lung diseases, eight keywords on mechanism emerged, which are inflammation, apoptosis, NRF2, antioxidant, hypoxia, lipid peroxidation, mitochondria, NF-κB and autophagy. To explore the trend of scientists’ research focus, we analyzed the occurrence of these keywords in the publications on oxidative stress and pulmonary diseases during different time periods. As shown in Figure 2, except for antioxidant, hypoxia, and lipid peroxidation, the occurrences of these keywords during 2018–2022 increased by more than 1.5 times compared with those in 1953–2007. The occurrence of three keywords, including inflammation, NRF2 and NF-κB, increased dramatically. Obviously, inflammation remains a focus of interest, and the latter two keywords have emerged as hot spots of pulmonary research.

### Bubble diagrams demonstrate the frequency, connection, and citation numbers of the keywords visually during different time periods

**FIGURE 3:**
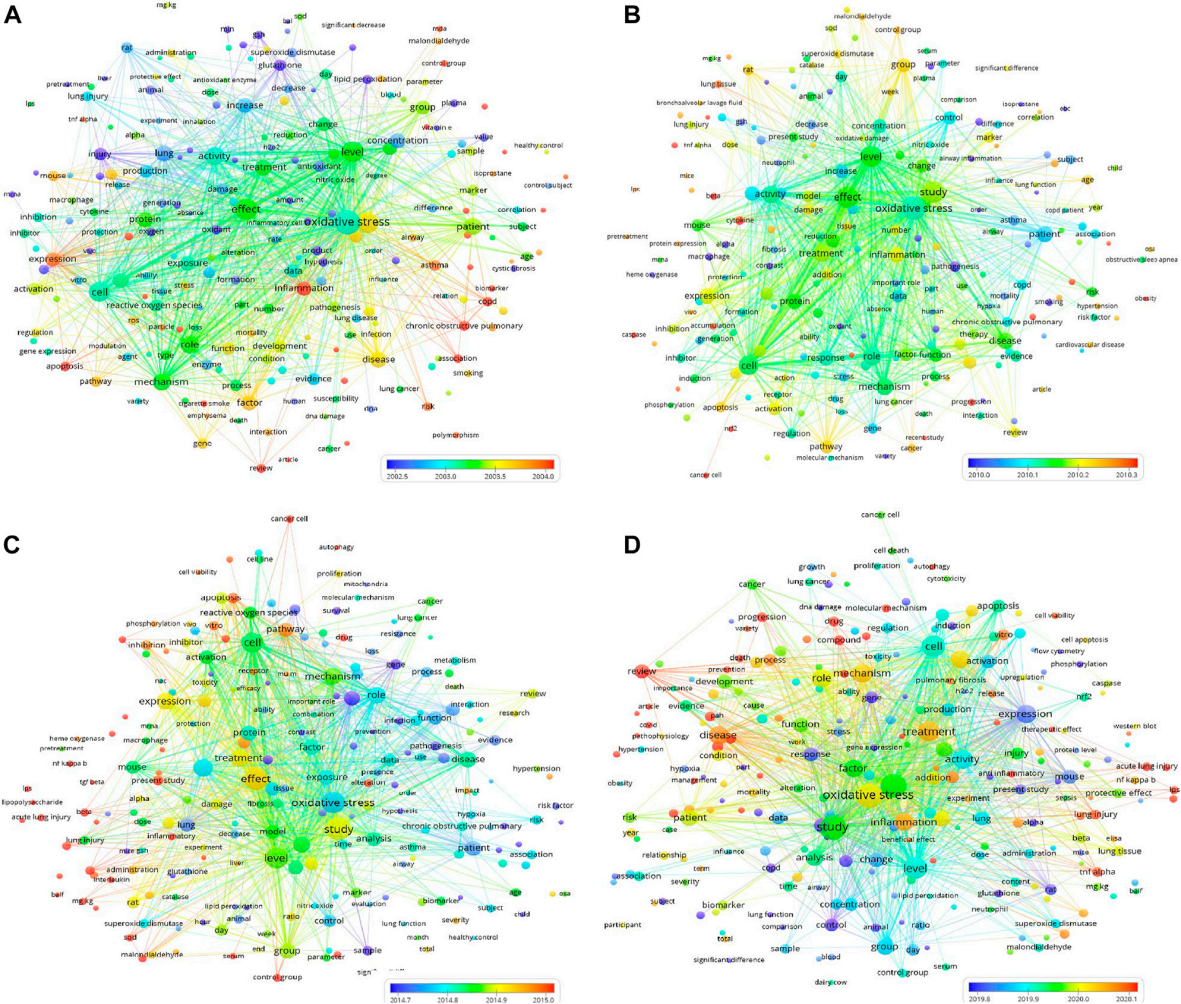
Four bubble diagrams representing the keywords appearing in the titles or abstracts of at least 2% publications on oxidative stress and lung diseases and their co-occurrence from 1953 to 2007, 2008 to 2012, 2013 to 2017, and 2018 to 2022, respectively. During the four periods, there are 228 [**(A)** from 1953 to 2007], 200 [**(B)**, from 2008 to 2012], 227 [**(C)** from 2013 to 2017] and 258 [**(D)** 2018–2022] bubbles in 3 clusters, respectively. The overlay score was demonstrated by rainbow color style as indicated. The above data can be viewed and downloaded from VOSviewer1.6.18 and this bubble diagram does not show all the terms and required data.

Furthermore, to visualize the frequency and connection of the keywords (words or phrases appearing in the titles or abstracts of the publications), the publications during four periods from 1953 to 2007, 2008 to 2012, 2013 to 2017, and 2018 to 2022 were imported into VOSviewer1.6.18 for all keyword analyses. As shown in Figures 3A–D, each keyword appearing in at least 2% of publications during the period was presented as a bubble in the bubble diagrams during the four time periods. The bubble size represents the frequency of its appearance in different publications during the time period (multiple occurrences of the word or phrase in one publication do not have repeat counts). The bubble color represents the overlay score among the keywords. In the bubble diagrams in Figures 3A–D, 228, 200, 227 and 258 terms appeared in the titles or abstracts of at least 2% of the publications on oxidative stress and pulmonary diseases from 1953 to 2007, 2008 to 2012, 2013 to 2017 and 2018 to 2022, respectively.

As shown in Figures 3A–D, the top 2% keywords with the highest average citation rates during all four time periods can be sorted into several clusters. The center words of the clusters include oxidative stress, lung, disease, injury, patients, factor, study, effect, mechanism, mouse, cell, inflammation, apoptosis, level, and expression. In Figure 3D, it is noted that COVID (*n* = 17, 2018–2022) appeared in the titles or abstracts of at least 2% of the publications, suggesting a rapid increase in the number of publications during this period. Moreover, N-acetylcysteine (*n* = 107, 1953–2007) and vitamin E (*n* = 128, 1953–2007) appeared as keywords in at least 2% of the publications, suggesting their acknowledged therapeutic effects in some pulmonary diseases. However, no therapeutic agent appeared in at least 2% of publications during 2008–2022.

### The number of publications on major pulmonary diseases is increasing annually

It is well known that excess oxidative stress can cause inflammation (Duecker et al., 2018; Wiegman et al., 2020). It has also been reported that iron homeostasis is disrupted and mitochondrial structure and function are defective in some pulmonary diseases (Ghio et al., 2003; Reid et al., 2007; Philippot et al., 2014; Rangarajan et al., 2018; Yu et al., 2018). Therefore, we analyzed the number of publications that are related to iron, mitochondria, or inflammation by adding the retrieval conditions of TS = (“inflammat*”), TS=(“iron” or “Ferroptosis”), and TS=(“mitochondria” or “mitochondrial” or “mitochondrion”), respectively, to the retrieval pattern of the previously retrieved 31,373 publications in the WOS electronic databases.

**FIGURE 4:**
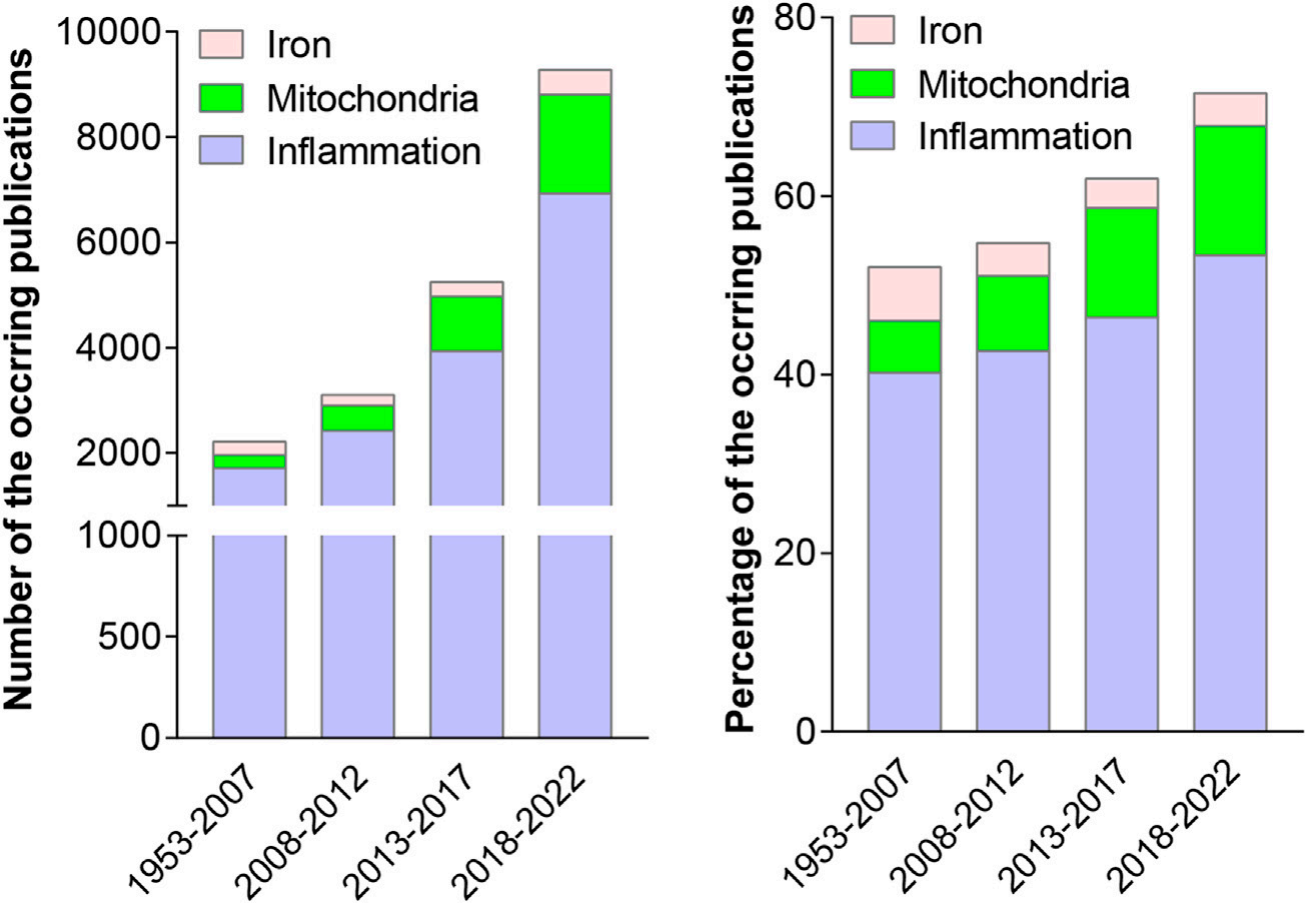
Numbers of the retrieved publications on oxidative stress and lung diseases that are related to iron, mitochondria, and inflammation (the left panel), and their percentages (the right panel) were analyzed during the four periods, from 1953 to 2007, 2008 to 2012, 2013 to 2017 and 2018 to 2022.

As shown in Figure 4, both the total number (1710/1953–2007, 2,419/2008–2012, 3,932/2013–2017, 6,923/2018–2022) and the proportion (40.2%/1953–2007, 42.6%/2008–2012, 46.4%/2013–2017, 53.4%/2018–2022) of publications related to inflammation showed obvious increasing trends during each time period. Similarly, publications related to mitochondria demonstrated an elevation in both the number (248/1953–2007, 479/2008–2012, 1,040/2013–2017, 1884/2018–2022) and the proportion (5.8%/1953–2007, 8.4%/2008–2012, 12.3%/2013–2017, 14.5%/2018–2022) of publications during each time period. In addition, the proportion (6.0%/1953–2007, 3.6%/2008–2012, 3.2%/2013–2017, 3.6%/2018–2022) of iron-related publications first decreased and then increased during the four periods.

**FIGURE 5:**
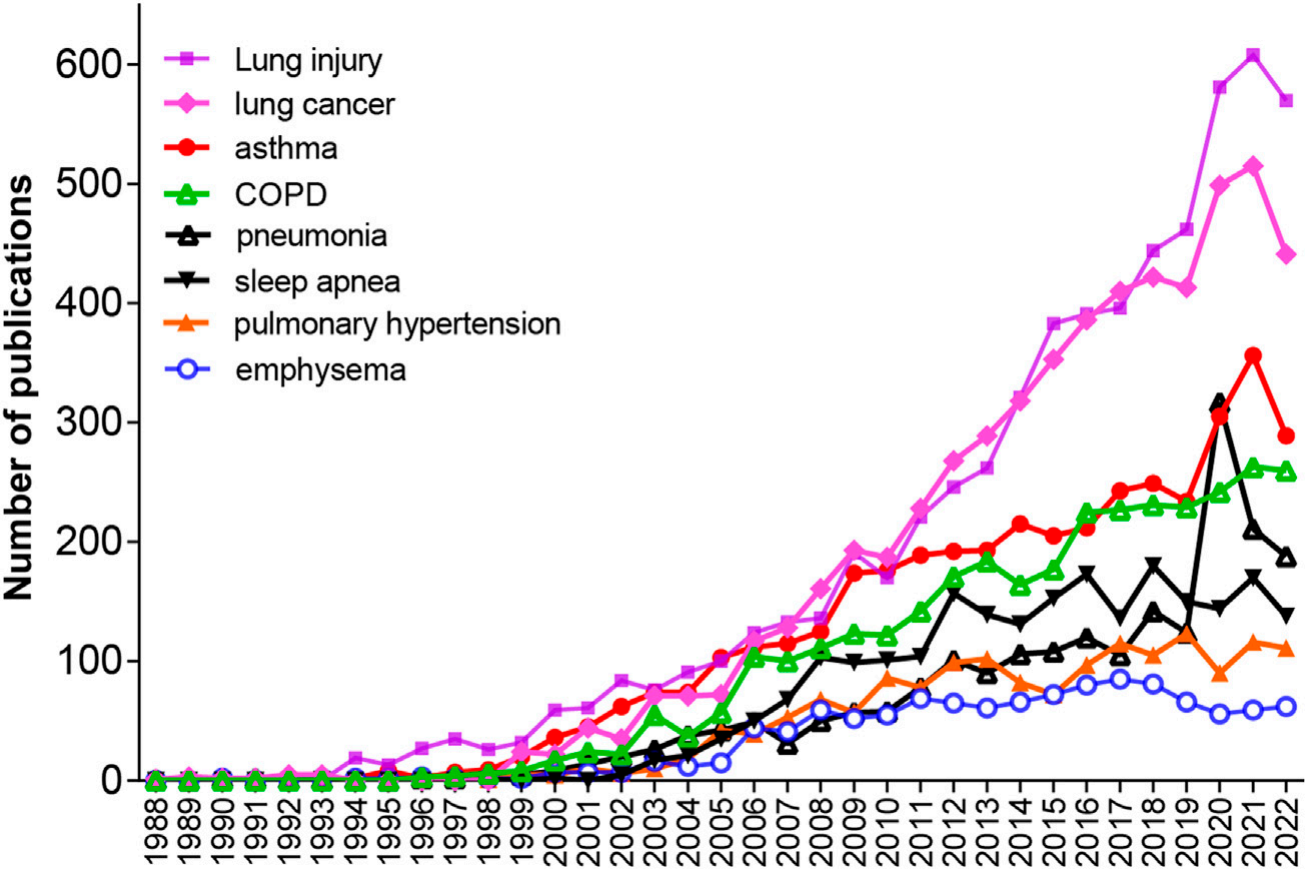
Annual publications on eight pulmonary diseases, including lung injury, lung cancer, asthma, COPD, pneumonia, sleep apnea, pulmonary hypertension and emphysema, respectively, from 1953 to 2022.

To explore the numbers of publications on each kind of pulmonary disease related to oxidative stress during 1953–2022, we performed another advanced search in the WOS electronic databases by using the retrieval words of TS = (“Oxidative Stress*”) AND TS = (“the corresponding disease”). As shown in Table 4, among the 33 lung diseases, the top 8 diseases with the most publications were lung injury (6,285/20.0%), lung cancer (5,725/18.2%), asthma (4,034/12.9%), COPD (3,311/10.6%), sleep apnea (2,278/7.3%), pneumonia (2,106/6.7%), pulmonary hypertension (1,606/5.1%), and emphysema (1,156/3.7%). Figure 5 shows that between 1988 and 2021, the annual number of publications regarding these pulmonary diseases has been continuously growing rapidly in general except for those on emphysema, and the number of publications on lung injury showed the fastest growing trend, followed by those on lung cancer and asthma. There was a drop in 2022, which may have been caused by shifted scientist interests to COVID-19 and the prevalence of COVID-19.

**TABLE 4:** The number of publications on oxidative stress and different categories of lung diseases from 1953 to 2022.

Disease term	Publication number/percentage	Disease term	Publication number/percentage
lung injury	6,285/20.0%	pleural effusion	335/1.1%
lung cancer	5,725/18.2%	dyspnea	230/0.7%
asthma	4,034/12.9%	lung edema	193/0.6%
COPD	3,311/10.6%	lung infection	139/0.4%
sleep apnea	2,278/7.3%	pneumoconiosis	118/0.4%
pneumonia	2,106/6.7%	bronchiectasis	86/0.3%
pulmonary hypertension	1,606/5.1%	alveolitis	81/0.3%
emphysema	1,159/3.7%	bronchiolitis obliterans syndrome	40/0.1%
cystic fibrosis	1,155/3.7%	alveolar proteinosis	37/0.1%
acute respiratory distress syndrome	775/2.5%	pneumothorax	16/0.1%
lung fibrosis	660/2.1%	lung tuberculosis	4/0.01%
hypoxemia	509/1.6%	ventilation dysfunction	3/0.01%
bronchitis	444/1.4%	lung sarcoidosis	2/0.01%
respiratory failure	433/1.4%	lung abscess	2/0.01%

### Potential therapeutic drugs for pulmonary diseases are summarized

By keyword analysis of the 31,373 publications on oxidative stress and pulmonary diseases between 1953 and 2022, the top 30 most frequent drugs are shown in Table 5. The top 10 medicines included N-acetylcysteine (*n* = 458/1.46%), vitamin E (n = 382/1.22%), vitamin C (*n* = 303/0.97%), dexamethasone (*n* = 225/0.72%), curcumin (*n* = 216/0.69%), β-carotene (*n* = 180/0.57%), polyphenols (*n* = 149/0.47%), carotenoid (*n* = 118/0.38%), vitamin D (*n* = 100/0.32%) and sulforaphane (*n* = 89/0.28%), indicating their close associations with oxidative stress and their potential use as therapeutics for pulmonary diseases. Furthermore, we analyzed the number of publications on each of the top eight pulmonary diseases (Table 5). The appearance of N-acetylcysteine had the most publications related to COPD (*n* = 81/2.45%), followed by lung cancer (*n* = 62/1.08%) and lung injury (*n* = 53/0.84%). Vitamin E appeared most in publications related to asthma (*n* = 66/1.64%), followed by lung cancer (*n* = 64/1.12%) and lung injury (*n* = 22/0.35%). Vitamin C has the most appearances in publications related to lung cancer (*n* = 70/1.22%), followed by asthma (*n* = 50/1.24%) and pneumonia (*n* = 30/1.43%). Dexamethasone appeared most in publications related to asthma (*n* = 100/2.48%). Notably, N-acetylcysteine appeared the most frequently in publications on lung injury, COPD, pneumonia, sleep apnea and emphysema; beta-carotene, dexamethasone, and sildenafil appeared the most frequently in publications on lung cancer, asthma and lung hypertension, respectively.

**TABLE 5:** The top thirty drugs appearing most in 31,373 publications on oxidative stress and pulmonary diseases as keywords from 1953 to 2022 were presented, and the number of their occurring publications and percentages on oxidative stress and each of the eight lung diseases were shown, respectively. Eight pulmonary diseases include lung injury, lung cancer, asthma, COPD, sleep apnea, pneumonia, pulmonary hypertension and emphysema.

Compound (n/percentage)	Lung injury	Lung cancer	Asthma	COPD	Sleep apnea	Pneumonia	Lung hypertension	Emphysema
N-acetylcysteine (458/1.46%)	53/0.84%	62/1.08%	49/1.21%	81/2.45%	20/0.88%	38/1.8%	9/0.56%	20/1.73%
vitamin E (382/1.22%)	22/0.35%	64/1.12%	66/1.64%	21/0.63%	6/0.26%	12/0.57%	19/1.18%	7/0.6%
vitamin C (303/0.97%)	14/0.22%	70/1.22%	50/1.24%	25/0.76%	8/0.35%	30/1.43%	12/0.75%	8/0.69%
Dexamethasone (225/0.72%)	41/0.65%	10/0.17%	100/2.48%	34/1.03%	3/0.13%	17/0.81%	2/0.12%	11/0.95%
Curcumin (216/0.69%)	30/0.48%	68/1.19%	23/0.57%	17/0.51%	1/0.04%	13/0.62%	2/0.12%	4/0.35%
Beta-carotene (180/0.57%)	41/0.65%	84/1.47%	31/0.77%	8/0.24%	2/0.09%	17/0.33%	1/0.06%	3/0.26%
Polyphenol (149/0.47%)	12/0.19%	43/0.75%	19/0.47%	13/0.39%	3/0.13%	14/0.66%	2/0.12%	6/0.52%
Carotenoid (118/0.38%)	1/0.02%	75/1.31%	17/0.42%	9/0.27%	1/0.04%	1/0.05%	1/0.06%	1/0.09%
vitamin D (100/0.32%)	6/0.1%	10/0.17%	31/0.77%	19/0.57%	4/0.18%	16/0.76%	1/0.06%	6/0.52%
Sulforaphane (89/0.28%)	12/0.19%	26/0.45%	9/0.22%	9/0.27%	2/0.09%	6/0.28%	3/0.19%	10/0.86%
Lycopene (80/0.25%)	2/0.03%	31/0.54%	15/0.37%	6/0.18%	0	1/0.05%	0	4/0.35%
Apocynin (79/0.25%)	9/0.14%	3/0.05%	19/0.47%	9/0.27%	9/0.4%	10/0.47%	7/0.44%	3/0.26%
Sildenafil (63/0.2%)	3/0.05%	0	1/0.02%	0	0	1/0.05%	42/2.62%	1/0.09%
Retinol (63/0.2%)	4/0.06%	19/0.33%	49/0.22%	4/0.12%	2/0.09%	2/0.09%	1/0.06%	0
Taurine (57/0.18%)	9/0.14%	31/0.02%	9/0.22%	5/0.15%	3/0.13%	2/0.09%	1/0.06%	3/0.26%
Rutin (57/0.18%)	18/0.29%	9/0.16%	9/0.22%	2/0.06%	1/0.04%	6/0.28%	1/0.06%	0
Theophylline (51/0.16%)	41/0.02%	62/0.03%	22/0.55%	37/1.12%	0	2/0.09%	1/0.06%	6/0.52%
Simvastatin (42/0.13%)	8/0.13%	7/0.12%	4/0.1%	4/0.12%	0	2/0.09%	6/0.37%	3/0.26%
Erdosteine (40/0.13%)	6/0.1%	31/0.02%	3/0.07%	26/0.79%	0	3/0.14%	0	6/0.52%
Budesonide (40/0.13%)	4/0.06%	3/0.05%	23/0.57%	22/0.66%	0	3/0.14%	0	1/0.09%
Edaravone (39/0.12%)	13/0.21%	0	1/0.02%	2/0.06%	2/0.09%	1/0.05%	1/0.06%	2/0.17%
vitamin A (35/0.11%)	1/0.02%	5/0.09%	5/0.12%	2/0.06%	2/0.09%	1/0.05%	1/0.06%	1/0.09%
Silymarin (32/0.1%)	4/0.06%	7/0.12%	3/0.07%	2/0.06%	0	3/0.14%	0	0
Nigella sativa (31/0.1%)	4/0.06%	5/0.09%	15/0.37%	1/0.03%	0	1/0.05%	0	1/0.09%
Berberine (30/0.1%)	5/0.08%	6/0.1%	0	1/0.03%	0	0	5/0.31%	0
Gefitinib (29/0.09%)	0	26/0.45%	0	0	0	1/0.05%	0	0
Apigenin (28/0.09%)	4/0.06%	10/0.17%	1/0.02%	1/0.03%	0	1/0.05%	0	0
Montelukast (26/0.08%)	2/0.03%	1/0.02%	16/0.4%	0	1/0.04%	2/0.09%	0	0
Baicalin (25/0.08%)	5/0.08%	1/0.02%	2/0.05%	4/0.12%	0	2/0.09%	2/0.12%	0
Crocin (23/0.07%)	3/0.05%	1/0.02%	5/0.12%	4/0.12%	0	2/0.09%	0	0

## Discussion

Oxidative stress has been reported to play a critical role in the pathology of multiple pulmonary diseases (Cheresh et al., 2013; Kirkham and Barnes, 2013; van der Vliet et al., 2018). Since 1983, the number of publications on oxidative stress and pulmonary diseases have continued to increase in general, and the cumulative number of publications is 31,373 until 2022. Unfortunately, quantitative and qualitative bibliometric analyses of the literature on oxidative stress and pulmonary diseases are lacking. This review presented a comprehensive and quantitative analysis of all the literature on oxidative stress and pulmonary disease. The United states, China, Italy, Japan, and England are the top five countries with the most publications from 1953 to 2022. The information published during four separate periods, 1953–2007, 2008–2012, 2013–2017, and 2018–2022, was also shown to demonstrate the developing trend. The increasing trend in the annual number of publications from 1983 to 2022, in addition to the large volume, demonstrates the close relationship between oxidative stress and pulmonary diseases, which is leading to growing scientific interest.

The common symptoms of lung diseases are cough, sputum, hemoptysis, and dyspnea. The causes of lung diseases vary and include air pollution, smoking, and viral and bacterial infections, which trigger or are closely related to oxidative stress in the lung. Notably, electronic cigarettes and heated tobacco products also showed harmful effects, including impaired pulmonary function and airway remodeling, and increased risk of cardiac and neurological diseases (Bahl et al., 2012; Lippi et al., 2014; Bhatt et al., 2020; Gu et al., 2023). As the lung is in direct contact with ultrafine particles in the air, it is challenging and critical to maintain redox homeostasis in the lung. In this study, it was demonstrated that oxidative stress has broad associations with the pathology of most pulmonary diseases, with the most publications in lung injury, lung cancer, asthma, COPD, and pneumonia, demonstrating the close connections between oxidative stress and these five diseases and the strong interest of scientists in these diseases. Mechanistically, oxidative stress causes cell signaling changes, organelle dysfunction, and alterations in the functions and fate of multiple cell types and leads to pathological conditions in the lung.

Among the keywords and the frequent keywords in the bubble diagrams, inflammation, mitochondria, autophagy, ER (endoplasmic reticulum) stress, apoptosis, DNA damage, hypoxia, vascular endothelial growth factor (VEGF), NRF2, and NF-κB demonstrate the most studied mechanisms of oxidative stress and pulmonary diseases. Inflammation appeared in 40%–60% of publications from 4 time periods as keywords. Continuous oxidative stress is acknowledged to cause chronic inflammation, which mediates most chronic diseases, including cancer, diabetes, cardiovascular and neurological diseases (Reuter et al., 2010; Hussain et al., 2016). In the lung, oxidative stress increases inflammation, at least partly through attenuated macrophage function (Flohe et al., 1997; Mokhtari-Zaer et al., 2020).

Mitochondria play a critical role in maintaining normal cell function. Excessive mitochondrial ROS increase the permeability of the mitochondrial membrane, oxidize the sulfhydryl group of enzymes, and cause irreversible damage to mitochondrial DNA, resulting in electron transport chain impairment and mitochondrial membrane potential loss (Liu and Chen, 2017). Mitochondrial dysfunction then contributes to cell abnormalities, such as hyperproliferation in airway smooth muscle cells or apoptosis in alveolar epithelial cells (Wiegman et al., 2015; Yu et al., 2018).

Iron imbalance and especially iron accumulation have been reported in several pulmonary diseases, including chronic obstructive pulmonary disease, cystic fibrosis, pulmonary alveolar proteinosis, and lung fibrosis (Ghio et al., 2008; Neves et al., 2019). The iron chelator deferoxamine alleviates bleomycin-induced lung fibrosis in a mouse model (Ali et al., 2020), indicating that pulmonary iron accumulation contributes to the pathology of pulmonary fibrosis. Iron can produce excessive hydroxyl radicals through the Fenton reaction (Lu and Koppenol, 2005), and the accumulation of excessive ROS promotes mitochondrial dysfunction (Gu et al., 2019). In addition, when free radicals attack unsaturated fatty acids on cell membranes, lipid peroxidation occurs. Severe lipid peroxidation can initiate ferroptosis, which is characterized by iron-dependent lipid peroxidation, metabolic constraints and abnormal mitochondrial morphology, including condensed mitochondrial membrane densities, smaller mitochondrial volume, diminished or vanished mitochondrial cristae, and ruptured mitochondrial outer membrane, and is regulated by glutathione peroxidase 4 (Seibt et al., 2019; Wang et al., 2020). Ferroptosis of cells was reported to play a role in the pathology of COPD, lung injury and lung cancer, and a ferrostatin inhibitor alleviated LPS-induced acute lung injury (Alvarez et al., 2017; Li et al., 2019; Yoshida et al., 2019; Liu et al., 2020).

Autophagy is a catabolic intracellular pathway that plays a crucial role in maintaining cellular homeostasis. Autophagy is associated with allergen-induced oxidative stress and regulates cell senescence (Sachdeva et al., 2019). Mitophagy was reported to reduce EMT (epithelial-mesenchymal transition) induced by TGF-β1, thus inhibiting the progression of pulmonary fibrosis (Prashanth Goud et al., 2019). Additionally, oxidative stress and endoplasmic reticulum stress interact with each other directly and indirectly in respiratory disease (Chen et al., 2018).

Oxidative stress can trigger DNA damage and lung epithelial cell death in pulmonary fibrosis (Cheresh et al., 2013), and mediates lung damage caused by intermittent hypoxia (Tuleta et al., 2016). During the oxidative stress induced by cerebral ischemia/reperfusion injury, the hypoxia inducible factor 1 subunit alpha (HIF-1α)/VEGF signaling pathway is activated, enabling repair of the endothelial barrier in the lung; additionally, the NRF2/heme oxygenase-1 (HO-1) pathway is activated to upregulate antioxidative stress (Fan et al., 2019).

NRF2 is a member of the Cap “n” Collar (CNC) family ofbasic leucine zipper transcription factors and is a “master regulator” of the antioxidant response in the cell. NRF2 regulates the expression of hundreds of genes, including antioxidant enzymes (SOD, catalase, and phase II detoxifying enzymes), genes participating in immune and inflammatory responses, tissue remodeling and fibrosis and carcinogenesis and metastasis (Martin et al., 1998; Itoh et al., 1999; Hybertson et al., 2011). Kelch-like ECH-associated protein 1 (KEAP1) negatively regulates NRF2 by targeting NRF2 for proteasomal degradation; conversely, NRF2 can drive KEAP1 transcription (Singh et al., 2006; Tian et al., 2020). The NRF2-KEAP1 system is an evolutionarily conserved intracellular defense mechanism that counteracts oxidative stress (Bellezza et al., 2018). HO-1 is a phase II detoxifying enzyme transcriptionally regulated by NRF2 and participates in the degradation of heme into biliverdin, carbon monoxide (CO), and Fe^2+^. Through these metabolites, the NRF2/HO-1 axis exerts protective roles against oxidation, apoptosis and ferroptosis in an organism (Zhang et al., 2018; Fan et al., 2019; Dong et al., 2020; Huang et al., 2020). NRF2 was reported to attenuate the progression of pulmonary fibrosis by inhibiting EMT mediated by suppressing snail expression and upregulating Numb (Zhou et al., 2016; Zhang et al., 2018). Moreover, NRF2 has been suggested as a therapeutic target for lung oxidative injuries, cancer, Alzheimer’s disease, and diabetic cardiomyopathy (Lu et al., 2016; Dianat et al., 2018; Bahn and Jo, 2019; Ge et al., 2019).

As a transcription factor, NF-κB plays essential roles in inflammatory responses. Oxidative status activates NF-κB activity by causing the dissociation of NF-κB from its inhibitor, IκB (inhibitor of NF-κB), and facilitating its nuclear translocation (Flohe et al., 1997). NF-κB overexpression is related to COPD, and it regulates the release of inflammatory mediators such as interleukin 1 beta (IL1β), C-X-C motif chemokine ligand 8 (CXCL8) and cytochrome c oxidase subunit II (COX2) in COPD (Zhou et al., 2018).

**FIGURE 6:**
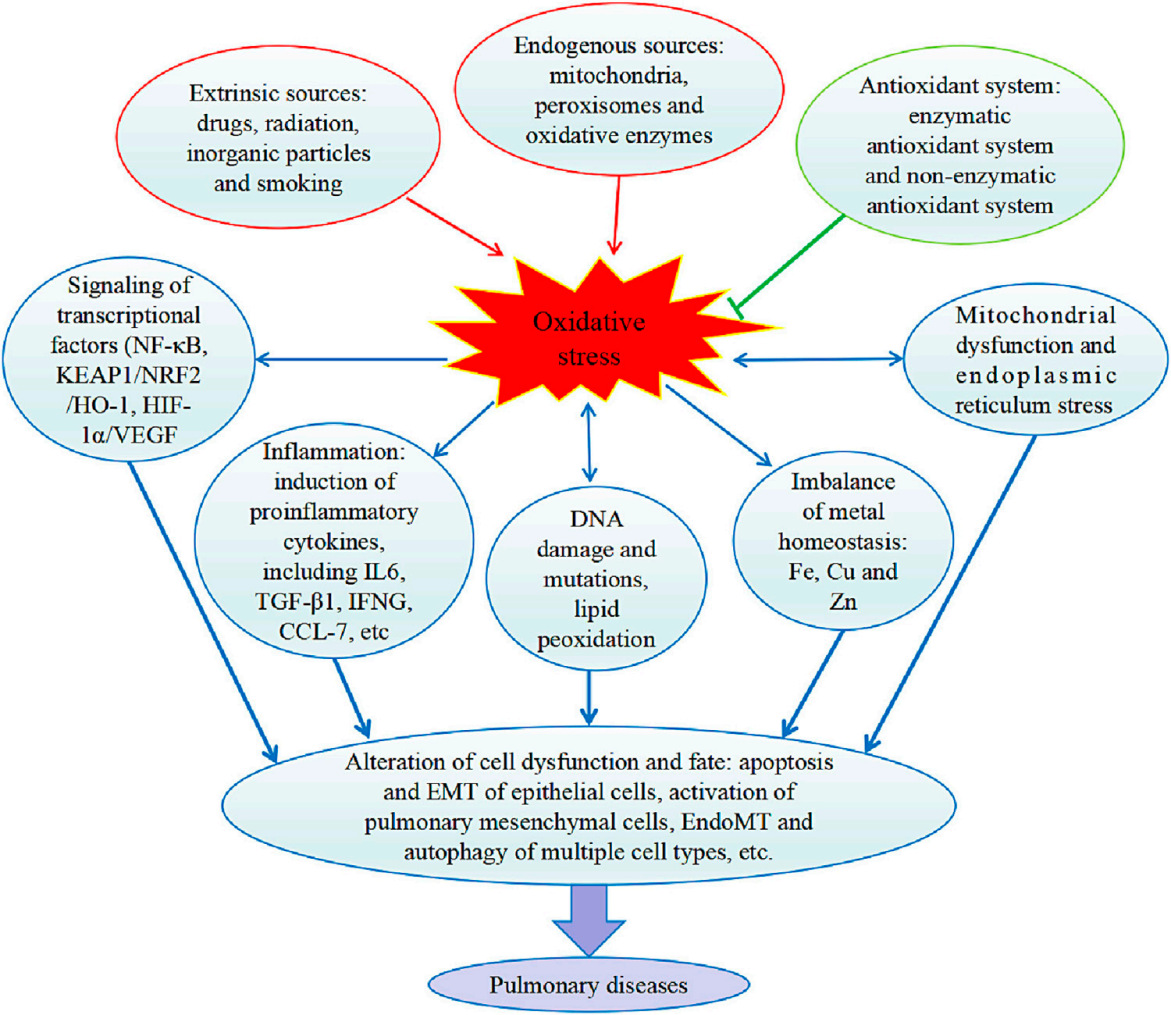
Internal and external causes of oxidative stress and effects of oxidative stress on the pathology of pulmonary diseases. In the lung, production of ROS (reactive oxygen species) by endogenous factors and extrinsic factors enhances oxidative stress and antioxidant system suppresses oxidative stress. Oxidative stress triggers the cell signaling pathways mediated by activation of transcriptional factors, and inflammatory cytokines were induced. Oxidative stress also damages DNA and lipids, and disturbs homeostatic balance of metals. Then, the resulted organelle normal function was defected, e.g., mitochondrial dysfunction and ER (endoplasmic reticulum) stress occur. Collectively, a series of cell malfunctions was induced, including apoptosis and EMT (epithelial-mesenchymal transition) of epithelial cells, activation of pulmonary mesenchymal cells, EndoMT (endothelial to mesenchymal transition) and autophagy of multiple cell types. IL6, interleukin 6. TGF-β1, transforming growth factor beta 1. IFNG, Interferon-gamma. CCL-7, chemokine C-C motif ligands 7. NF-κB, nuclear factor-κB. KEAP1, Kelch-like ECH-associated protein. NRF2, nuclear factor, erythroid 2 like 2. HO-1, heme oxygenase-1. HIF-1α, hypoxia inducible factor 1 subunit alpha. VEGF, vascular endothelial growth factor.

The generation of oxidative stress and the pathological processes resulting in pulmonary diseases are summarized in Figure 6. It should be noted that oxidative stress leads to different lung diseases with some similarities and distinct features. For example, most of the pathological mechanisms of pulmonary diseases involve inflammation. While COPD is characterized by excessive airway epithelial cell death and lung injury, lung cancer is caused by uncontrolled epithelial cell proliferation (Goldkorn et al., 2014). Some patients with asthma and COPD show overlapping features, which may be because of the cooccurrence of the two diseases or the different phenotypes of each disease (Barnes, 2017). In addition, for patients with age-related fibrotic diseases such as COPD and lung fibrosis, targeting autophagy and mitochondrial dysfunction that are related to metabolic homeostasis may offer better interventions than other strategies. Collectively, targeting the specific mechanisms underlying a particular pulmonary disease is necessary for developing truly effective therapeutics.

## Conclusions and future directions

In this review, thirty potential therapeutic drugs are discussed. Vitamin E helps remove free radicals and regulates the immune system and inflammation (Lewis et al., 2019). In addition to being an antioxidant, N-acetylcysteine is a mucolytic agent and can be used to treat respiratory diseases caused by excessive secretion of thick mucus (Raghu et al., 2020). Polyphenols inhibit the expression of inducible nitric oxide synthase and protect against oxidative and nitroxidative lung injury (Impellizzeri et al., 2015). Several polyphenol compounds, including resveratrol, curcumin, isorhamnetin, tannic acid, and honokiol, can attenuate pulmonary fibrosis (Conte et al., 2015; Amini et al., 2018; Zheng et al., 2019; Pulivendala et al., 2020; Rajasekar et al., 2020; Wang et al., 2022). The effects of these potential therapeutic agents have been determined mainly in experimental animals; however, confirmation of their effects requires more evidence from the bedside.

At present, there is no truly effective medicine that can be used to cure several common pulmonary diseases, such as lung cancer, COPD, and lung fibrosis. It is evident that some kinds of pulmonary diseases share similarities in pathways, and therapeutics, although each lung disease is unique. It is well recognized that ROS and RNS are both deleterious and beneficial species for physiological processes (Qu et al., 2011). Therefore, in the light of the origin, targeting harmful ROS or RNS species in specific organelles with nanotechniques, in combination with regulation of critical upstream signaling pathways and improvement of important downstream pathological processes, such as mitochondrial dysfunction, ferroptosis and inflammation through combined medication, in a particular pulmonary disease, will be a promising direction to cure these diseases.
